# Association of Prepregnancy Cardiovascular Risk Factors Clusters With Stillbirth Risk Across Racial and Ethnic Groups: A Nationwide Population‐Based Study of 31.4 Million Singleton Births and 131 047 Stillbirths

**DOI:** 10.1161/JAHA.124.042319

**Published:** 2025-07-29

**Authors:** Jing Nie, Leandro F. M. Rezende, Gerson Ferrari, Yuan Qiu, Xiaoling Wang, Wentao Huang, Zhiping Niu, Xiong Chen, Dagfinn Aune

**Affiliations:** ^1^ Population Research Institute, Nanjing University of Posts and Telecommunications Nanjing Jiangsu China; ^2^ High‐Quality Development Evaluation Research Institute, Nanjing University of Posts and Telecommunications Nanjing Jiangsu China; ^3^ Jiangsu High‐Quality Development Comprehensive Evaluation Research Base Nanjing University of Posts and Telecommunications Nanjing Jiangsu China; ^4^ Chronic Disease Epidemiology Research Center, Department of Preventive Medicine, Escola Paulista de Medicina Universidade Federal de São Paulo Sao Paulo Brazil; ^5^ Facultad de Ciencias de la Salud Universidad Autónoma de Chile Providencia Chile; ^6^ Universidad de Santiago de Chile (USACH), Escuela de Ciencias de La Actividad Física, El Deporte y La Salud Santiago Chile; ^7^ Department of Wound Repair The First Affiliated Hospital of Wenzhou Medical University Wenzhou Zhejiang China; ^8^ Department of Endocrinology The First Affiliated Hospital of Wenzhou Medical University Wenzhou Zhejiang China; ^9^ Postanesthsia Care Unit, Department of Anesthesiology, State Key Laboratory of Oncology in South China, Guangdong Key Laboratory of Nasopharyngeal Carcinoma Diagnosis and Therapy, Guangdong Provincial Clinical Research Center for Cancer Sun Yat‐sen University Cancer Center Guangzhou China; ^10^ Department of Nursing, State Key Laboratory of Oncology in South China, Guangdong Key Laboratory of Nasopharyngeal Carcinoma Diagnosis and Therapy, Guangdong Provincial Clinical Research Center for Cancer Sun Yat‐sen University Cancer Center Guangzhou China; ^11^ Department of Environmental Health, School of Public Health Fudan University Shanghai China; ^12^ Wenzhou Key Laboratory of Diabetes Research Wenzhou Zhejiang China; ^13^ Department of Epidemiology and Biostatistics, School of Public Health Imperial College London London United Kingdom; ^14^ Department of Nutrition Oslo New University College Oslo Norway; ^15^ Department of Research Cancer Registry of Norway, Norwegian Institute of Public Health Oslo Norway

**Keywords:** cardiovascular health, epidemiology, pregnancy, risk factor, stillbirth, Epidemiology, Pediatrics, Pregnancy, Risk Factors

## Abstract

**Background:**

The relationship between specific clusters of prepregnancy cardiovascular risk factors and stillbirth, particularly across racial and ethnic groups, remains understudied. We aimed to evaluate the association between 16 distinct cardiovascular risk clusters and stillbirth, emphasizing racial disparities.

**Methods:**

We conducted a nationwide, population‐based study using Centers for Disease Control and Prevention Natality and Fetal Death Data Files (2014–2022), including 31 408 776 singleton births and 131 047 stillbirths (≥20 weeks' gestation). Prepregnancy cardiovascular risk factors—diabetes, hypertension, smoking, and nonideal body mass index—were categorized into 16 mutually exclusive clusters. Modified Poisson regression with robust variance estimation was used to estimate adjusted relative risks and 95% CIs.

**Results:**

The analysis of the 16 groups defined by the 4 binary risk factors revealed that, compared with clusters without any risk factors, clusters with a single risk factor, absent the other 3, displayed varying degrees of stillbirth risk: prepregnancy diabetes posed the greatest risk (adjusted relative risk, 4.05 [95% CI, 3.74–4.39]), followed by prepregnancy hypertension (adjusted relative risk, 2.44 [95% CI, 2.27–2.63]), smoking (adjusted relative risk, 1.62 [95% CI, 1.58–1.67]), and unhealthy body mass index (adjusted relative risk, 1.33 [95% CI, 1.31–1.35]). The absolute excess risk attributable to biological interaction between 2 risk factors was observed in 2 of 6 risk combinations, predominantly those involving diabetes. Racial disparities were pronounced, with non‐Hispanic Black mothers exhibiting the highest relative and absolute risks of stillbirth, nearly double that of non‐Hispanic White mothers.

**Conclusions:**

Prepregnancy cardiovascular risk profiles are strongly associated with stillbirth, with marked racial and ethnic disparities. Simplified scoring systems may obscure heterogeneity in risk, reinforcing the need for race‐conscious, targeted interventions.

**Clinical Perspective**

**What Is New?**

- An increasing number of maternal cardiovascular risk factors were associated with higher risk of stillbirth, and subjects with all 4 risk factors had a >9‐fold increase in relative risk compared with those with no risk factors.
- Non‐Hispanic Black mothers exhibited both higher absolute and relative risks of stillbirth compared with Non‐Hispanic White, Hispanic, and other racial and ethnic groups; simplified risk scoring systems may obscure critical differences in risk profiles, underscoring the necessity for tailored interventions to address racial and ethnic disparities in stillbirth outcomes.

**What Are the Clinical Implications?**

- There is a pressing need for tailored recommendations or interventions aimed at high‐risk populations to mitigate racial and ethnic disparities in pregnancy outcomes.

Stillbirth, defined as the intrauterine death of a fetus typically occurring between 20 and 28 weeks of gestation, is widely recognized as a key indicator of the quality and effectiveness of health care systems during pregnancy and childbirth.^1^, ^2^, ^3^ It is estimated that ≈2 million stillbirths occurred globally in 2019.^3^ In the United States, the stillbirth rate for pregnancies beyond 20 weeks' gestation was 5.48 per 1000 live births and fetal deaths, totaling around 20 200 stillbirths in 2022. Although the stillbirth rate in the United States has declined over the past 3 decades, it remains higher than in many other high‐income countries, underscoring the need for further reduction and improvement in outcomes.^4^

Maternal prepregnancy cardiovascular health has been identified as a modifiable and preventable risk factor for stillbirth.^5^ Previous studies have demonstrated a significant positive association between the total burden of prepregnancy cardiovascular risk factors and stillbirth occurring after 20 weeks of gestation.^6^ However, it is unclear whether this association differs by race and ethnicity. Additionally, the prepregnancy cardiovascular risk score was constructed under the assumption that each risk factor contributes equally to the overall risk. As a result, individuals with different combinations of risk factors may experience varying levels of stillbirth risk. The specific risk associated with different combinations of these factors remain to be determined.

Addressing this issue is essential not only for enabling physicians to provide personalized advice and care but also for offering valuable insights to policymakers and public health officials in developing targeted, tailored health promotion strategies and interventions aimed at reducing stillbirth rates. Using a nationwide birth data set from the United States, we assessed the association between cumulative prepregnancy cardiovascular health risk factors (diabetes, hypertension, smoking, and an “unhealthy” body mass index [BMI]) and stillbirth risk. We also performed stratified and joint association analyses across varying racial and ethnic groups. Subsequently, we examined the association of 16 different combinations of these risk factors with stillbirth risk.

## METHODS

### Data Availability

All data shown in the main text and supplementary information are available from the corresponding author upon request.

### Study Population and Data Source

We conducted a nationwide population‐based retrospective cohort study using data from the Centers for Disease Control and Prevention National Center for Health Statistics, incorporating the Natality and Fetal Death Data Files (Fetal Death Files exclude all terminations), which encompass all live births and fetal deaths, covering the period from January 1, 2014, to December 31, 2022 (https://www.cdc.gov/nchs/data_access/vitalstatsonline.htm). The detailed description of the data set can be found in previously published studies.^1^, ^2^, ^6^ A total of 34 755 636 live births and fetal deaths were identified during the study period. After excluding women with maternal age <15 or >49 years, non‐US residency, multiple births, and missing data on prepregnancy BMI, smoking, hypertension, and diabetes, the final analytical sample included 31 408 776 singleton births. Since the analyses were conducted using publicly available, deidentified data, ethical approval was not required for this study. We followed the Strengthening the Reporting of Observational Studies in Epidemiology guidelines in the preparation of this article.^7^

### Exposures

Consistent with previous studies,^6^ the cardiovascular health score first comprised 4 binary factors: hypertension, diabetes (without distinction between type 1 and type 2), unhealthy weight, and smoking. Each factor was assigned 1 point if present, resulting in a total score ranging from 0 to 4. Unhealthy weight was defined as a BMI outside the range of 18.5 to 24.99 kg/m^2^. Then, 4 binary factors were categorized into 16 distinct clusters based on the presence or absence of these factors.

### Outcomes

The definitions of stillbirth differ significantly among countries, with gestational age thresholds ranging from fetal death after 12 weeks to ≥28 weeks.^2^ In this study, we used 20 weeks in the United States. To enable comparison, we report results using 3 different definitions (≥20 weeks' gestation, ≥24 weeks' gestation, and ≥28 weeks' gestation). For all analyses, we used ≥20 weeks' gestation, with results using ≥24 and ≥28 weeks also reported for main analysis.

### Potential Confounders and Mediators

Confounders were selected on the basis of prior research where they are known or plausibly suspected to be common causes of both cardiovascular health risk factors and stillbirth, in line with best practices.^8^, ^9^ These include maternal age at birth (categorized into 6 groups: 15–19, 20–24, 25–29, 30–34, 35–39, and 40–49 years), maternal race and ethnicity (Hispanic, non‐Hispanic White, non‐Hispanic Black, and other [Non‐Hispanic American Indian or Alaska Native, Non‐Hispanic Asian, Non‐Hispanic Native Hawaiian or Other Pacific Islander, and Non‐Hispanic more than one race]) based on single/multiple‐race provided by public use files, maternal education level (<high school degree, high school degree and >high school degree).

Potential mediators could dilute the association between cardiovascular health risk factors and stillbirth. In this study, we did not adjust for potential mediators, including gestational clinical risk factors (gestational diabetes, gestational hypertension, and preeclampsia).

### Statistical Analysis

Data were analyzed from December 12, 2022, to February 20, 2025. We compared the characteristics of the analysis sample (complete cases) with the full data set to identify any patterns of missingness. Given the low proportion of missing data on confounder variables, the primary analysis was conducted using a complete case data set.

First, multivariable modified Poisson regression models with robust error variance were used to calculate adjusted relative risks (RRs) with corresponding 95% CIs for the association between prepregnancy cardiovascular score and stillbirth risk. Second, we conducted stratified analyses by race and ethnicity, using a likelihood ratio test to examine interaction terms. Additionally, we used similar likelihood ratio tests to evaluate interactions between maternal age and prepregnancy cardiovascular scores. We then conducted joint association analyses involving the 16 groups created by the 4 prepregnancy cardiovascular scores and 4 racial and ethnic groups, both in relation to stillbirth risk. Subsequently, we examined the joint association of the 16 groups defined by the 4 binary risk factors (diabetes, hypertension, smoking, and an unhealthy BMI) with stillbirth risk. The population attributable fraction was computed for each of the 16 groups.^10^ Finally, we only conducted a joint association analysis involving 32 groups, formed by combining 16 prepregnancy cardiovascular clusters and 2 racial and ethnic groups (non‐Hispanic White and non‐Hispanic Black), in relation to stillbirth. We excluded the Hispanic and other racial and ethnic groups from the analysis due to some risk factor clusters having <2 events. All adjusted stillbirth rates above were calculated using the postestimation margins command at average levels.

A series of sensitivity analyses were conducted to ensure the robustness of the main results. Multiple imputation using chained equations was used to address missing data on confounder variables. Given that missing data on prepregnancy cardiovascular risk factors could bias the primary association, we applied multiple imputation to manage missing data for both prepregnancy cardiovascular risk factors and confounder variables. Additionally, birth year was adjusted for in the main analysis. We also excluded underweight participants from the analysis. E‐values were computed for the primary analysis.^11^ A 2‐sided *P* value of <0.05 was deemed significant. All statistical analyses were conducted using StataSE 15.0 (StataCorp, College Station, TX).

## RESULTS

### Characteristics of the Study Population

A total of 31 408 776 singleton births including 131 047 (4.2/1000 births) stillbirths at or after 20 completed weeks between 2014 and 2022 were included in the complete case analysis sample (Table S1). The stillbirth rate was lower in the complete case sample compared with the whole sample (4.2 versus 5.3/1000 births), with the distribution of all other variables being similar (Table S1). Compared with those without prepregnancy cardiovascular risk factors, women with 3 or 4 risk factors were older and predominantly non‐Hispanic Black individuals (Table 1).

**Table 1:** Baseline Demographic Characteristics According to CVD Risk Factors

Variable	CVD risk factors*				
	0	1	2	3	4
No. of subjects (%)	12 969 660 (42.67)	16 288 648 (53.59)	2 029 243 (6.68)	115 540 (0.38)	5685 (0.02)
Maternal age, y, n (%)					
15–19	807 987 (51.34)	686 876 (43.64)	77 320 (4.91)	1720 (0.11)	34 (0.01)
20–24	2 491 680 (40.02)	3 264 019 (52.42)	456 937 (7.34)	13 688 (0.22)	416 (0.01)
25–29	3 562 831 (39.32)	4 854 036 (53.57)	614 951 (6.79)	27 480 (0.30)	1154 (0.01)
30–34	3 856 919 (42.87)	4 582 151 (50.94)	519 871 (5.78)	35 244 (0.39)	1849 (0.02)
35–39	1 867 569 (41.14)	2 356 669 (51.91)	285 796 (6.30)	28 100 (0.62)	1683 (0.04)
40–49	382 674 (37.82)	544 897 (53.85)	74 368 (7.35)	9308 (0.92)	549 (0.05)
Maternal race and ethnicity, n (%)					
Hispanic	2 776 842 (37.00)	4 467 737 (59.53)	245 504 (3.27)	14 692 (0.20)	410 (0.01)
Non‐Hispanic White	7 130 090 (43.27)	7 983 061 (48.44)	1 301 203 (7.90)	61 119 (0.37)	3245 (0.02)
Non‐Hispanic Black	1 439 679 (32.21)	2 654 522 (59.39)	343 448 (7.68)	30 568 (0.68)	1533 (0.03)
Other†	1 623 049 (54.92)	1 183 328 (40.04)	139 088 (4.71)	9161 (0.31)	497 (0.02)
Maternal education level, n (%)					
Less than high school degree	1 431 804 (35.67)	2 216 893 (55.23)	344 727 (8.59)	19 447 (0.48)	1139 (0.03)
High school degree	2 814 817 (34.64)	4 527 538 (55.71)	741 168 (9.12)	40 867 (0.50)	2155 (0.03)
More than high school degree	8 723 039 (45.27)	9 544 217 (49.53)	943 348 (4.90)	55 226 (0.29)	2391 (0.01)
History of cesarean section, n (%)	11 510 321 (43.26)	13 415 786 (50.42)	1 598 685 (6.01)	81 761 (0.31)	3756 (0.01)
No	1 459 339 (30.41)	2 872 862 (59.87)	430 558 (8.97)	33 779 (0.70)	1929 (0.04)
Yes	807 987 (51.34)	686 876 (43.64)	77 320 (4.91)	1720 (0.11)	34 (0.01)

CVD indicates cardiovascular disease.

* Cardiovascular risk factors included diabetes, hypertension, smoking, and nonnormal body mass index.

^†^ Non‐Hispanic American Indian or Alaska Native, Non‐Hispanic Asian, Non‐Hispanic Native Hawaiian or Other Pacific Islander, and Non‐Hispanic more than one race.

### Prepregnancy Cardiovascular Risk Factor Scores and Stillbirth Across Race and Ethnicity

**Figure 1:**
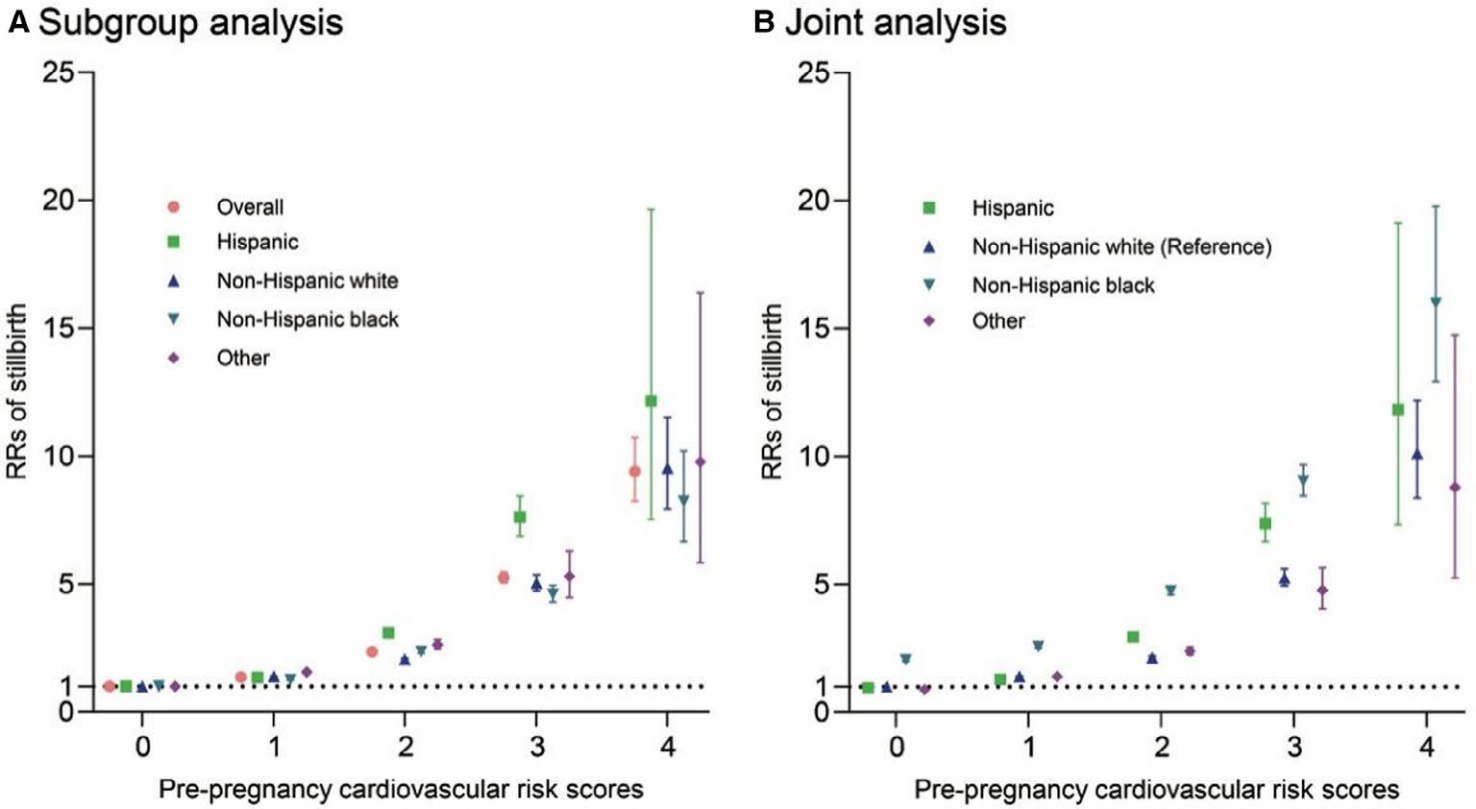
Association of prepregnancy cardiovascular risk scores and stillbirth across race and ethnicity. Subgroup analysis: Stratified analyses were conducted by race and ethnicity, with each of the 4 prepregnancy cardiovascular risk scores used as the exposure variable. Joint analysis: Stratified analyses were conducted using a composite exposure variable consisting of 16 mutually exclusive groups derived by cross‐classifying the 4 scores with the 4 racial and ethnic categories. RR indicates relative risk.

Higher cardiovascular risk factor scores were associated with an increased risk of stillbirth (risk factors: 1: RR, 1.36 [95% CI, 1.35–1.38]; 2: RR, 2.35 [95% CI, 2.30–2.39]; 3: RR, 5.25 [95% CI, 5.03–5.47]; 4: RR, 9.40 [95% CI, 8.25–10.72]; Table S2). The association between cardiovascular risk factor scores and stillbirth risk varied significantly by race and ethnicity (*P*_interaction_<0.001; Figure 1, Table S3). Across all racial and ethnic groups, RRs of stillbirth increased with higher prepregnancy cardiovascular risk factor scores (Figure 1); however, baseline risk was ≈2‐fold higher among non‐Hispanic Black women as illustrated by the joint analysis (Table S3).

In the joint analysis of 16 groups defined by 4 racial and ethnic categories and 4 cardiovascular risk factor scores, the magnitude of the association among non‐Hispanic Black mothers was approximately 50% to 100% higher than that of non‐Hispanic White mothers when mothers had the same cardiovascular risk factors. Among Hispanic mothers, the magnitude of the association for having 1 cardiovascular risk factor was lower compared with non‐Hispanic White mothers with the same risk factor. However, for those with ≥2 cardiovascular risk factors, the association was higher than that observed in non‐Hispanic White mothers. Similarly, in mothers from other racial and ethnic groups, the magnitude of the association with stillbirth risk varied significantly compared with non‐Hispanic White mothers with identical risk factors (Table S3).

Additionally, a significant interaction effect was observed between cardiovascular risk factor scores and maternal age on stillbirth risk (*P*_interaction_<0.001; Table S4).

### Prepregnancy Clusters of Cardiovascular Risk Factors and Stillbirth Risk

Further analysis exploring the relationship between each of the 16 prepregnancy risk factor clusters and stillbirth revealed that the presence of any single risk factor was associated with an increased risk of stillbirth compared with mothers with none of the 4 risk factors (Table 2). Women with only 1 risk factor, while the other 3 were absent, had a higher RR of stillbirth, but the magnitude of risk varied across the different risk factors. Compared with mothers without any of the 4 risk factors, diabetes posed the highest risk (RR, 4.05 [95% CI, 3.74–4.39]; predicted stillbirth rate, 12.2/1000 births [95% CI, 11.3–13.2]; Table 2), followed by hypertension (RR, 2.44 [95% CI, 2.27–2.63]; predicted stillbirth rate, 7.4/1000 births [95% CI, 6.9–7.9]); smoking (RR, 1.62 [95% CI, 1.58–1.67]; predicted stillbirth rate, 4.9/1000 births [95% CI, 4.8–5.0]), and unhealthy BMI (RR, 1.33 [95% CI, 1.31–1.35]; predicted stillbirth rate, 4.0/1000 births [95% CI, 4.0–4.1]), which presented the lowest risk among the 4 factors. The absolute excess risk due to biological interaction between 2 risk factors was observed in 2 of 6 combinations, primarily those involving diabetes. These combinations included: diabetes and smoking (absolute excess risk, 5.3/1000 births); diabetes and hypertension (absolute excess risk, 4.2/1000 births). The prepregnancy risk factor clusters that included a higher number of risk factors generally had higher RRs and stillbirth rates compared with those with fewer risk factors. However, mothers with the 3 risk factors of diabetes, hypertension, and smoking had similar RRs and stillbirth rates to those with all 4 risk factors (diabetes, hypertension, and smoking: RR, 9.46 [95% CI, 6.02–14.88] versus 4 risk factors: RR, 9.45 [95% CI, 8.29–10.78]). The cluster with a single prepregnancy unhealthy BMI while the other 3 were absent contributed to the highest proportion of stillbirths, with a population attributable fraction of 12.34%, which exceeds the combined population attributable fraction of the other 14 cluster combinations (11.20%). When using the 24‐ and 28‐week threshold definitions of stillbirth, the results showed some variation in certain risk factor clusters, such as prepregnancy unhealthy BMI and smoking, compared with the 20‐week definition of stillbirth (Table S5).

**Table 2:** Joint Association of Each CVD Risk Factor With Stillbirth Risk

Pregnancy CVD risk factors					Stillbirth	Participants	Stillbirth rate per 1000 births*	Difference in stillbirth rate (per 1000 births)	RR (95% CI)	E‐value (limit)†	PAF (%)
No.	Unhealthy BMI	Smoking	Hypertension	Diabetes							
0	0	0	0	0	39 542	12 969 660	3.02 (2.99–3.05)	0 (Reference)	1.00 (Reference)	…	…
1	1	0	0	0	65 199	15 021 222	4.01 (3.98–4.05)	1.00 (0.95–1.04)	1.33 (1.31–1.35)	1.99 (1.95)	12.34
	0	1	0	0	6036	1 133 085	4.90 (4.77–5.02)	1.88 (1.75–2.01)	1.62 (1.58–1.67)	2.62 (2.54)	1.77
	0	0	1	0	749	89 053	7.38 (6.85–7.90)	4.36 (3.83–4.89)	2.44 (2.27–2.63)	4.31 (3.97)	0.34
	0	0	0	1	605	47 288	12.23 (11.26–13.20)	9.21 (8.24–10.18)	4.05 (3.74–4.39)	7.57 (6.94)	0.35
2	1	1	0	0	8670	1 371 478	5.72 (5.59–5.84)	2.70 (2.57–2.83)	1.89 (1.85–1.94)	3.19 (3.10)	3.12
	1	0	1	0	4597	457 682	8.16 (7.92–8.40)	5.14 (4.90–5.38)	2.70 (2.62–2.79)	4.84 (4.68)	2.21
	1	0	0	1	2679	179 541	12.94 (12.45–13.43)	9.92 (9.43–10.41)	4.28 (4.12–4.46)	8.03 (7.71)	1.57
	0	1	1	0	182	12 321	11.71 (10.02–13.40)	8.69 (7.00–10.38)	3.88 (3.36–4.48)	7.22 (6.18)	0.10
	0	1	0	1	121	4910	22.41 (18.47–26.36)	19.39 (15.45–23.34)	7.42 (6.22–8.86)	14.32 (11.92)	0.08
	0	0	1	1	98	3311	23.76 (19.13–28.40)	20.75 (16.11–25.38)	7.87 (6.48–9.57)	15.22 (12.44)	0.07
3	1	1	1	0	761	53 981	11.07 (10.29–11.85)	8.05 (7.27–8.84)	3.67 (3.41–3.94)	6.80 (6.28)	0.42
	1	1	0	1	451	18 705	20.73 (18.84–22.62)	17.71 (15.82–19.61)	6.87 (6.26–7.53)	13.22 (12.00)	0.29
	1	0	1	1	1122	42 356	19.84 (18.68–20.99)	16.82 (15.67–17.98)	6.57 (6.20–6.97)	12.62 (11.88)	0.73
	0	1	1	1	18	498	28.56 (15.63–41.50)	25.55 (12.61–38.48)	9.46 (6.02–14.88)	18.41 (11.52)	0.01
4	1	1	1	1	217	5685	28.54 (24.81–32.27)	25.52 (21.79–29.25)	9.45 (8.29–10.78)	18.39 (16.06)	0.15

Adjustment for maternal age, maternal education, and birth year. BMI indicates body mass index (calculated as weight in kg divided by height in m^2^); CVD, cardiovascular disease; PAF, population attributable fraction; and RR, relative risk.

* Predicted rate based on average marginal effects.

^†^ E‐value for the 95% confidence limit closest to the null.

**Figure 2:**
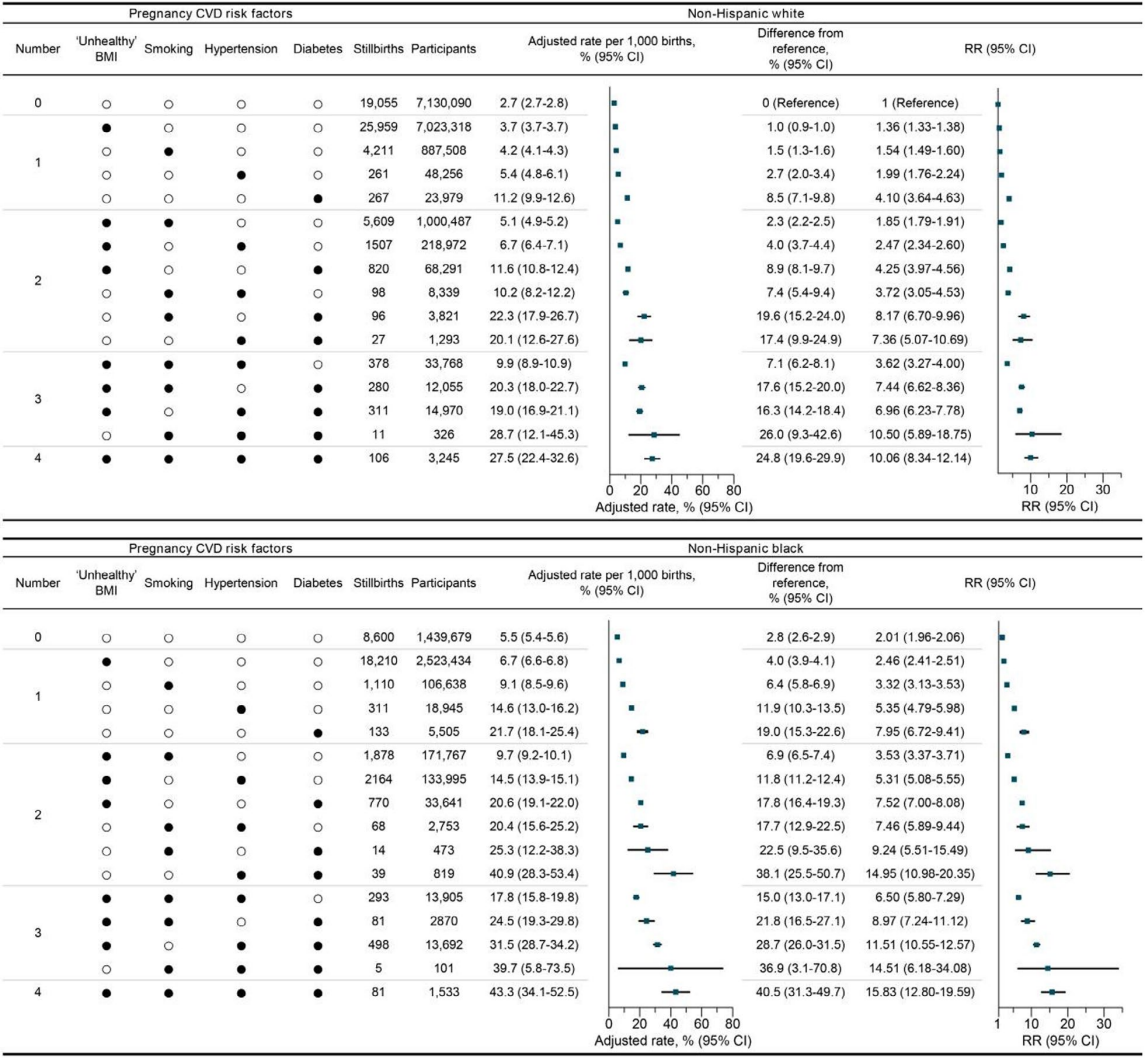
Joint association of 16 prepregnancy cardiovascular risk factor combinations with stillbirth risk across non‐Hispanic White and non‐Hispanic Black. BMI indicates body mass index (calculated as weight in kg divided by height in m^2^); CVD, cardiovascular disease; and RR, relative risk.

A subgroup analysis of the 16 cluster combinations for stillbirth risk was conducted separately for non‐Hispanic Black and White mothers (Figure 2, Table 3). Non‐Hispanic Black mothers who smoked or had hypertension while the other 2 risk factors were absent showed a higher magnitude of relative associations compared with non‐Hispanic White mothers, with those without any risk factors serving as the reference group in each racial group analysis (smoking while the other 3 risk factors were absent: non‐Hispanic Black mothers: RR, 1.71 [95% CI, 1.60–1.82]; non‐Hispanic White mothers: RR, 1.49 [95% CI, 1.44–1.55]; hypertension while the other 3 risk factors were absent: non‐Hispanic Black mothers: RR, 2.71 [95% CI, 2.42–3.04]; non‐Hispanic White mothers: RR, 1.97 [95% CI, 1.75–2.23]; Table 3). In the further joint analysis of 32 groups defined by 4 racial and ethnic categories and 16 cardiovascular risk factor clusters, non‐Hispanic Black mothers had the highest absolute stillbirth risk when compared with mothers with the same risk factor cluster, with their rate being approximately double that of non‐Hispanic White mothers without any of the 4 risk factors (predicted stillbirth rate: non‐Hispanic Black mothers: 5.5/1000 births [95% CI, 5.4–5.6]; non‐Hispanic White mothers: 2.7/1000 births [95% CI, 2.7–2.8]; Table 3, Figure 2). However, the magnitude of the differences between non‐Hispanic Black and non‐Hispanic White mothers varied somewhat across the different risk factor clusters (Table 3).

**Table 3:** Association of 16 Combinations With Stillbirth Risk Across Non‐Hispanic White and Non‐Hispanic Black

Pregnancy CVD risk factors					Stillbirth	Participants	Non‐Hispanic White	Stillbirth	Participants	Non‐Hispanic Black
No.	Unhealthy BMI	Smoking	Hypertension	Diabetes			RR (95% CI)			RR (95% CI)
0	0	0	0	0	19 055	7 130 090	1.00 (reference)	8600	1 439 679	1.00 (reference)
1	1	0	0	0	25 959	7 023 318	1.35 (1.32–1.37)	18 210	2 523 434	1.23 (1.19–1.26)
	0	1	0	0	4211	887 508	1.49 (1.44–1.55)	1110	106 638	1.71 (1.60–1.82)
	0	0	1	0	261	48 256	1.97 (1.75–2.23)	311	18 945	2.71 (2.42–3.04)
	0	0	0	1	267	23 979	4.09 (3.63–4.61)	133	5505	4.01 (3.39–4.75)
2	1	1	0	0	5609	1 000 487	1.80 (1.74–1.85)	1878	171 767	1.81 (1.72–1.90)
	1	0	1	0	1507	218 972	2.44 (2.31–2.57)	2164	133 995	2.69 (2.56–2.82)
	1	0	0	1	820	68 291	4.19 (3.91–4.49)	770	33 641	3.81 (3.54–4.10)
	0	1	1	0	98	8339	3.56 (2.92–4.34)	68	2753	3.92 (3.10–4.97)
	0	1	0	1	96	3821	7.90 (6.48–9.63)	14	473	4.79 (2.85–8.03)
	0	0	1	1	27	1293	7.24 (4.99–10.51)	39	819	7.70 (5.66–10.48)
3	1	1	1	0	378	33 768	3.48 (3.14–3.85)	293	13 905	3.39 (3.02–3.80)
	1	1	0	1	280	12 055	7.15 (6.36–8.03)	81	2870	4.61 (3.72–5.72)
	1	0	1	1	311	14 970	6.76 (6.05–7.56)	498	13 692	5.92 (5.41–6.47)
	0	1	1	1	11	326	9.96 (5.58–17.77)	5	101	7.69 (3.28–18.07)
4	1	1	1	1	106	3245	9.55 (7.91–11.52)	81	1533	8.31 (6.71–10.29)

Adjustment for maternal age, maternal education, and birth year. BMI indicates body mass index (calculated as weight in kg divided by height in m^2^); CVD, cardiovascular disease; and RR, relative risk.

### Sensitivity Analysis

Adjusting for birth year and previous cesareans, as well as excluding underweight subjects, did not substantially alter the main findings (Table S6). Analyses using multiple imputation to account for missing data on the 4 cardiovascular risk factors yielded estimates that were largely similar to those in the primary analysis. However, most of the estimates were slightly smaller after addressing the missing data on these risk factors, which may be explained by the fact that the proportion of missing cardiovascular risk factor data was more pronounced in the stillbirth data set. The E‐values indicate that an unmeasured confounder with a risk association of at least 1.99‐fold would be required to nullify the observed RR of 1.33, while an unmeasured confounder with an RR of 18.39 would be required to nullify the observed RR of 9.45 comparing mothers with all 4 risk factors to those with no risk factors, underscoring the robustness of the primary findings (Figure 2).

## DISCUSSION

In this population‐based study of >31.4 million multiethnic pregnant mothers, we found strong positive associations between maternal prepregnancy cardiovascular risk factors and increased risk of stillbirth. When compared with clusters without any risk factors, those with a single risk factor while the other 3 were absent demonstrated that prepregnancy diabetes presented the greatest relative risk, followed by prepregnancy hypertension, smoking, and, finally, suboptimal BMI. These findings highlight the importance of considering multifactorial interactions in risk assessments, as simplified scoring systems, while convenient, may obscure critical nuances in risk distribution among different populations. Despite “nonideal” BMI having a relatively lower RR compared with the other 3 risk factors, it accounted for more than half of the excess stillbirths across all risk clusters because of its high prevalence within the population. Furthermore, non‐Hispanic Black mothers had both higher absolute and relative stillbirth risk than non‐Hispanic White, Hispanic, and other racial and ethnic groups.

### Comparison With Other Studies

Consistent with previous studies,^2^, ^6^ we found that cardiovascular risk factor scores and each individual risk factor were associated with an increased risk of stillbirth. However, assigning similar point values to each risk factor metric is somewhat arbitrary,^12^ as demonstrated by our joint analysis of the 4 risk factors in this study. Due to the potential for complex multiplicative and additive interactions among different risk factors, the overall risk in groups with a total score of 1 is primarily driven by the specific risk factor with the highest prevalence or proportion in that group.^13^, ^14^, ^15^, ^16^ This dynamic limits the comparability of risk across different subgroup populations with varying distributions of risk factors.^17^

To reduce the overall population burden of stillbirth, the analysis of 16 cardiovascular risk factor clusters highlights prepregnancy unhealthy BMI as a key area for intervention, as it accounted for more than half of the excess stillbirth cases attributable to these risk factors. This finding is consistent with a large body of evidence linking higher maternal BMI with increased risk of a wide range of adverse pregnancy outcomes, including maternal diabetes, hypertension, and congenital anomalies, as well as stillbirths.^18^, ^19^, ^20^ It is also likely that maternal diabetes and hypertension or their preclinical precursors are on the causal pathway between excess weight and stillbirth. Given the strong associations between maternal BMI and these risk factors,^18^ the number of mothers with isolated diabetes or isolated hypertension was relatively low compared with the groups with the combination of unhealthy BMI and diabetes or hypertension. From an individual health risk perspective, mothers with prepregnancy diabetes should receive enhanced care and better management of other risk factors, as the combination of diabetes with additional factors can significantly increase the risk of stillbirth. A comprehensive approach that includes both managing prepregnancy diabetes and addressing associated cardiovascular risk factors is essential to mitigate this elevated risk and improve maternal and neonatal outcomes.

Previous studies have evaluated ethnic disparities in adverse pregnancy outcomes,^8^, ^21^, ^22^ and a meta‐analysis reported higher risks of stillbirth and other pregnancy complications among infants born to Black women.^23^ Another study demonstrated that the association between low Apgar scores and infant death varies across racial and ethnic groups, revealing that this association is weaker among non‐Hispanic Black infants compared with their non‐Hispanic White and Hispanic counterparts.^22^ Similarly, our study using a 4‐risk‐factor score found that the association between a single‐risk‐factor score and stillbirth, in comparison with the absence of any risk factors, was stronger among non‐Hispanic White, Hispanic, and other racial and ethnic groups than among non‐Hispanic Black individuals, which can be explained by the higher absolute stillbirth risk among non‐Hispanic Black mothers. The RRs for stillbirth associated with scores of 1, 2, 3, and 4 did not show a consistent pattern across non‐Hispanic White and non‐Hispanic Black racial and ethnic groups, which may be attributed to the differing distribution and composition of risk factors among these populations, which are supported by our analysis using a combination of 16 risk factors across racial and ethnic groups and some degree of chance variation due to low numbers in some clusters.^24^ Non‐Hispanic Black mothers with prepregnancy hypertension or smoking while the other 3 risk factors were absent demonstrated significantly higher relative and absolute stillbirth risks compared with non‐Hispanic White mothers. Although isolated unhealthy weight was positively associated with stillbirth risk in offspring of both non‐Hispanic Black and White mothers, the association was slightly stronger in White mothers.

Previous research has elucidated the association between maternity care, prenatal care quality, and exposure to environmental risk factors and the risk of stillbirth.^25^, ^26^, ^27^, ^28^ Notably, studies have demonstrated that experiences of discrimination during maternity care and mistreatment during pregnancy are more prevalent among non‐Hispanic Black women compared with their non‐Hispanic White counterparts.^29^, ^30^ Furthermore, non‐Hispanic Black mothers are disproportionately concentrated in counties with higher Maternal Vulnerability Index scores, reflecting greater exposure to community‐level structural disadvantages.^28^ These racial disparities in maternity care experiences, prenatal care quality, and community vulnerability may partially account for the persistent racial inequities observed in stillbirth rates.^31^

In our study, we demonstrated the complex associations between individual risk factors and health outcomes, as well as some heterogeneity in the effects of different combinations of multiple factors. These findings show that simple scoring systems of cardiovascular risk factors remain highly valuable in both clinical and public health settings, as they offer an intuitive and easily interpretable measure of cardiovascular health.^12^

### Strengths and Limitations

Our study has several key strengths. First, we used a nationally representative data set with a large sample size, which strengthens the generalizability of the findings across broader population subgroups. Second, the low proportion of missing data on confounder variables helps minimize potential bias in the analysis. Moreover, the large sample size provides sufficient statistical power to explore the joint effects of 16 multiple risk factor combinations on stillbirth outcomes. This is particularly important, as smaller sample sizes may not capture sufficient events within certain risk factor combinations, limiting the ability to conduct analyses in such scenarios. Finally, the large data set also enabled subgroup analyses across different racial and ethnic groups.

Our study also has several limitations. First, both smoking status and BMI were self‐reported rather than objectively measured, which may have introduced misclassification bias. For instance, individuals with higher BMI may underreport their weight, a common issue in self‐reported data, leading to potential misclassification of exposure. However, several studies suggest self‐reported weight and height^32^, ^33^, ^34^, ^35^ is highly correlated with measured weight and height as well as other measures of body fat,^36^, ^37^ suggesting any bias due to self‐report is likely to be small. Other confounders may also have been subject to measurement errors. However, given that we did observe strong positive associations between all included risk factors and stillbirth risk, consistent with available data from other cohorts,^20^, ^38^, ^39^, ^40^ the self‐reported data are likely to have some validity. Second, our study was limited by the available data on risk factors. It did not include information on other important risk factors, such as non–high‐density lipoprotein cholesterol, physical activity, diet, and sleep. Additionally, the 4 risk factors included in the analysis were dichotomized, consistent with previous studies, due to the way the score was operationalized as well as data limitations (eg, blood pressure and fasting blood glucose were not measured). Future research should aim to explore the combined impact of additional cardiovascular risk factors on stillbirth risk. Third, although the mechanisms underlying increased risk likely differ between underweight and overweight/obesity categories, the results remained similar when excluding the underweight category. The number of underweight participants was relatively small compared with the full cohort, and therefore the adverse effects of unhealthy BMI on stillbirth risk appeared to be mostly driven by the overweight and obese categories. Fourth, although the proportion of missing data on confounding variables was low and we performed multiple imputation to enhance the robustness of the results, the proportion of missing data for risk factors was not negligible and not at random. Therefore, despite our imputation approach, some degree of bias may remain. Fifth, due to database limitations, we were unable to account for other important confounders, such as income, occupation, community‐level factors, and parity. Although E‐values indicated that potential unmeasured or residual confounding may be less likely to fully explain away the observed associations as the E‐values were high, we cannot completely rule out the possibility that unmeasured confounding could have led to under‐ or overestimation of the associations. Finally, this study was unable to explore the mechanisms underlying the observed associations. Given differences in population characteristics, the generalizability of our findings to other countries should be interpreted with caution. Future research should validate these findings using data from other countries to assess their broader applicability.

## CONCLUSIONS

In conclusion, our study showed a significant association between prepregnancy cardiovascular risk factor scores, individual risk clusters, and the risk of stillbirth in a nationwide US cohort. By combining 4 binary risk factors into 16 distinct risk distinct clusters, we found that the strength of association with stillbirth varied across clusters, with certain single risk factors (without the presence of other factors) showing stronger associations. Notably, prepregnancy diabetes posed the highest risk, followed by prepregnancy hypertension, smoking, and suboptimal BMI. This finding highlights the potential limitations of simplified scoring systems, which may obscure critical differences in how individual risk profiles influence health outcomes. Furthermore, we observed strong associations between risk clusters and stillbirth across different racial and ethnic groups, with both the absolute and relative risk of stillbirth being highest among non‐Hispanic Black mothers. These results underscore the need for tailored recommendations or interventions aimed at high‐risk populations, in particular, but also at all levels of the risk spectrum to help mitigate racial and ethnic disparities in pregnancy outcomes. These results provide support for recommendations and health policies to reduce the prevalence of cardiovascular risk factors among women of reproductive age.

## Sources of Funding

This study was supported by the National Council for Scientific and Technological Development (311109‐2023‐3; L.F.M.R.), the National Natural Science Foundation of China (82270364), and The Summit Advancement Disciplines of Zhejiang Province (Wenzhou Medical University–Pharmaceutics).

## Disclosures

None.

## Supporting information

Tables S1–S6
