# Supplementary material for: Association of Prepregnancy Cardiovascular Risk Factors Clusters With Stillbirth Risk Across Racial and Ethnic Groups: A Nationwide Population‐Based Study of 31.4 Million Singleton Births and 131 047 Stillbirths
Source: J Am Heart Assoc. 2025 Jul 29;14(15):e042319. doi: 10.1161/JAHA.124.042319 (PMC12449984; doi:10.1161/JAHA.124.042319)
Supplement: Supplementary file 1 — Tables S1–S6 [file JAH3-14-e042319-s001.pdf]

SUPPLEMENTAL MATERIAL

Table S1. The distribution of all other variables for whole sample and analysis sample.

Variable	Whole sample	Analysis sample
No of subjects, n	32,963,979	31,408,776
Stillbirth, n (rates per 1,000 births)	176,200 (5.3 per 1000 births)	131,047 (4.2 per 1000 births)
CVD risk factors*, %		
0	41.36	41.29
1	52.84	51.86
2	6.42	6.46
3	0.37	0.37
4	0.02	0.02
Maternal age, years, n (%)		
15-19	5.02	5.01
20-24	19.77	19.82
25–29	28.75	28.85
30–34	28.63	28.64
35–39	14.55	14.45
40-49	3.28	3.22
Maternal race/ethnicity, n (%)		
Hispanic	24.09	23.90
Non-Hispanic White	52.03	52.47
Non-Hispanic Black	14.41	14.23
Other	9.47	9.41
Maternal education level, n (%)		
Less than high school degree	13.00	12.78

High school degree	25.98	25.87
More than high school degree	61.03	61.35
History of cesarean section, n (%)		
Yes	15.29	15.28

* Cardiovascular risk factors included diabetes, hypertension, smoking and non-normal BMI.

Table S2. Association of pregnancy CVD risk factors with stillbirth risk.

Pregnancy CVD risk factors	Stillbirth	Participants	Crude model RR (95% CI)	Fully adjusted model RR (95% CI)	Sensitivity analysis model RR (95% CI)
0	39,542	12,969,660	1.00 (reference)	1.00 (reference)	1.00 (reference)
1	72,589	16,288,648	1.46 (1.44-1.48)	1.36 (1.35-1.38)	1.37 (1.36-1.39)
2	16,347	2,029,243	2.64 (2.59-2.69)	2.35 (2.30-2.39)	2.38 (2.33-2.42)
3	2,352	115,540	6.68 (6.41-6.96)	5.25 (5.03-5.47)	5.36 (5.14-5.58)
4	217	5,685	12.52 (10.98-14.27)	9.40 (8.25-10.72)	9.63 (8.45-10.98)

Fully adjusted model: adjusted for maternal age, maternal race/ethnicity, maternal education level and birthyear.

Sensitivity analysis model: additionally adjusted for previous cesareans.

Table S3. Association of pregnancy CVD risk factors with stillbirth across race and ethnicity.

Pregnancy CVD risk factors	Hispanic RR (95% CI)	Non-Hispanic white RR (95% CI)	Non-Hispanic black RR (95% CI)	Other ethnic groups RR (95% CI)
Participants, number	7,505,185	16,478,718	4,469,750	2,955,123
Stillbirth, number	28,068	58,996	34,255	9,728
Subgroup analysis				
0	**1.00 (reference)**	**1.00 (reference)**	**1.00 (reference)**	**1.00 (reference)**
1	1.35 (1.32 - 1.39)	1.38 (1.35 - 1.40)	1.26 (1.23 - 1.29)	1.56 (1.49 - 1.63)
2	3.09 (2.95 - 3.23)	2.06 (2.00 - 2.11)	2.37 (2.29 - 2.46)	2.63 (2.45 - 2.83)
3	7.60 (6.84 - 8.44)	5.03 (4.72 - 5.36)	4.60 (4.30 - 4.94)	5.29 (4.46 - 6.28)
4	12.15 (7.52 - 19.64)	9.54 (7.91 - 11.51)	8.24 (6.65 - 10.20)	9.77 (5.83 - 16.38)
Joint associations				
0	0.95 (0.93 - 0.98)	**1.00 (reference)**	2.04 (1.99 - 2.09)	0.89 (0.86 - 0.92)
1	1.29 (1.26 - 1.31)	1.40 (1.37 - 1.42)	2.56 (2.51 - 2.61)	1.40 (1.35 - 1.44)
2	2.95 (2.83 - 3.09)	2.13 (2.08 - 2.19)	4.74 (4.59 - 4.89)	2.39 (2.24 - 2.55)
3	7.38 (6.66 - 8.19)	5.25 (4.93 - 5.60)	9.04 (8.46 - 9.67)	4.77 (4.03 - 5.64)
4	11.83 (7.32 - 19.12)	10.12 (8.39 - 12.20)	15.98 (12.91 - 19.77)	8.80 (5.25 - 14.73)

Table S4. Association of pregnancy CVD risk factors with stillbirth across age.

Pregnancy CVD risk factors	***15-19*** RR (95% CI)	***20-24*** RR (95% CI)	***25-29*** RR (95% CI)	***30-34*** RR (95% CI)	***35-39*** RR (95% CI)	***40-49*** RR (95% CI)
Participants, number	1,573,937	6,226,740	9,060,452	8,996,034	4,539,817	1,011,796
Stillbirth, number	8,236	26,638	34,935	33,753	20,707	6,778
Subgroup analysis						
0	**1.00 (reference)**	**1.00 (reference)**	**1.00 (reference)**	**1.00 (reference)**	**1.00 (reference)**	**1.00 (reference)**
1	1.21 (1.15-1.26)	1.24 (1.2-1.27)	1.34 (1.31-1.38)	1.46 (1.42-1.49)	1.5 (1.45-1.55)	1.42 (1.34-1.51)
2	1.64 (1.5-1.79)	1.85 (1.77-1.93)	2.21 (2.13-2.29)	2.74 (2.64-2.84)	2.9 (2.77-3.03)	2.66 (2.45-2.88)
3	3.99 (2.82-5.63)	5.02 (4.44-5.68)	4.98 (4.54-5.45)	5.82 (5.39-6.29)	5.98 (5.51-6.49)	4.49 (3.91-5.15)
4	10.88 (2.86-41.36)	7.83 (4.59-13.34)	10.92 (8.2-14.55)	10.86 (8.64-13.65)	9.09 (7.09-11.65)	8 (5.42-11.8)

Table S5. Joint association of each CVD risk factors with Stillbirth using the 24-week and 28-week thresholds*.

Pregnancy CVD risk factors					Stillbirth (20-week) RR (95% CI)	Stillbirth†	Participants†	Stillbirth (24-week) RR (95% CI)	Stillbirth‡	Participants‡	Stillbirth (28-week) RR (95% CI)
Number	'Unhealthy' BMI	Smoking	Hypertension	Diabetes							
0	0	0	0	0	1.00 (Reference)	25994	12956112	1.00 (Reference)	19884	12950002	1.00 (Reference)
1	1	0	0	0	1.33 (1.31-1.35)	41686	14997709	1.30 (1.28-1.32)	32278	14988301	1.32 (1.30-1.34)
	0	1	0	0	1.62 (1.58-1.67)	4163	1129212	1.65 (1.60-1.71)	3259	1128308	1.67 (1.61-1.74)
	0	0	1	0	2.44 (2.27-2.63)	489	88793	2.46 (2.25-2.69)	326	88630	2.15 (1.93-2.40)
	0	0	0	1	4.05 (3.74-4.39)	495	47178	5.05 (4.62-5.52)	423	47106	5.63 (5.12-6.20)
2	1	1	0	0	1.89 (1.85-1.94)	6102	1368910	1.99 (1.93-2.04)	4844	1367652	2.04 (1.98-2.11)
	1	0	1	0	2.70 (2.62-2.79)	2896	455981	2.65 (2.55-2.75)	1930	455015	2.33 (2.22-2.44)
	1	0	0	1	4.28 (4.12-4.46)	2034	178896	5.02 (4.80-5.25)	1768	178630	5.70 (5.43-5.99)
	0	1	1	0	3.88 (3.36-4.48)	128	12267	4.14 (3.48-4.92)	88	12227	3.71 (3.01-4.57)
	0	1	0	1	7.42 (6.22-8.86)	95	4884	8.68 (7.11-10.60)	74	4863	8.77 (7.00-11.01)
	0	0	1	1	7.87 (6.48-9.57)	61	3274	7.61 (5.93-9.76)	47	3260	7.71 (5.80-10.24)
3	1	1	1	0	3.67 (3.41-3.94)	556	53776	4.09 (3.76-4.45)	380	53600	3.66 (3.31-4.05)
	1	1	0	1	6.87 (6.26-7.53)	378	18632	8.70 (7.87-9.63)	331	18585	9.89 (8.88-11.02)
	1	0	1	1	6.57 (6.20-6.97)	784	42018	7.21 (6.71-7.74)	600	41834	7.28 (6.71-7.89)
	0	1	1	1	9.46 (6.02-14.88)	12	492	9.60 (5.50-16.75)	9	489	9.40 (4.93-17.94)
4	1	1	1	1	9.45 (8.29-10.78)	167	5635	11.24 (9.67-13.06)	142	5610	12.49 (10.61-14.7)

BMI indicates body mass index (calculated as weight in kilograms divided by height in meters squared); CVD, cardiovascular disease; RR, relative risk.

* aRRs were adjusted for maternal age, race/ethnicity, education level and birth year;

† the number of participants and stillbirth events for the outcome of stillbirth using 24 weeks;

‡ the number of participants and stillbirth events for the outcome of stillbirth using 28 weeks.

Tables S6. Sensitivity analysis for joint association of each CVD risk factors with stillbirth risk.

Pregnancy CVD risk factors					Main analysis	Sensitivity analysis 1 RR (95% CI)	Sensitivity analysis 2 RR (95% CI)	Sensitivity analysis 3 RR (95% CI)	Sensitivity analysis 4 RR (95% CI)
Number	'Unhealthy' BMI	Smoking	Hypertension	Diabetes					
0	0	0	0	0	1.00 (Reference)	1.00 (Reference)	1.00 (Reference)	1.00 (Reference)	1.00 (Reference)
1	1	0	0	0	1.33 (1.31-1.35)	1.34 (1.32-1.36)	1.31 (1.29-1.32)	1.33 (1.31-1.34)	1.33 (1.32-1.35)
	0	1	0	0	1.62 (1.58-1.67)	1.63 (1.58-1.68)	1.51 (1.46-1.55)	1.58 (1.54-1.62)	1.64 (1.59-1.69)
	0	0	1	0	2.44 (2.27-2.63)	2.45 (2.28-2.64)	2.40 (2.25-2.56)	2.47 (2.30-2.64)	2.40 (2.23-2.58)
	0	0	0	1	4.05 (3.74-4.39)	4.1 (3.78-4.44)	3.97 (3.69-4.28)	4.02 (3.73-4.34)	4.01 (3.69-4.35)
2	1	1	0	0	1.89 (1.85-1.94)	1.92 (1.87-1.96)	1.78 (1.74-1.82)	1.84 (1.80-1.89)	1.90 (1.85-1.94)
	1	0	1	0	2.7 (2.62-2.79)	2.74 (2.66-2.83)	2.67 (2.60-2.74)	2.69 (2.62-2.77)	2.71 (2.63-2.8)
	1	0	0	1	4.28 (4.12-4.46)	4.38 (4.21-4.55)	4.20 (4.06-4.35)	4.28 (4.13-4.45)	4.30 (4.13-4.47)
	0	1	1	0	3.88 (3.36-4.48)	3.91 (3.38-4.52)	3.50 (3.03-4.04)	3.87 (3.37-4.44)	3.86 (3.32-4.49)
	0	1	0	1	7.42 (6.22-8.86)	7.56 (6.33-9.01)	6.95 (5.94-8.14)	7.50 (6.34-8.87)	7.37 (6.13-8.87)
	0	0	1	1	7.87 (6.48-9.57)	8 (6.58-9.73)	7.20 (6.03-8.61)	7.98 (6.64-9.59)	7.60 (6.21-9.31)
3	1	1	1	0	3.67 (3.41-3.94)	3.73 (3.47-4.01)	3.55 (3.34-3.78)	3.64 (3.40-3.90)	3.68 (3.43-3.95)
	1	1	0	1	6.87 (6.26-7.53)	7.04 (6.42-7.71)	6.25 (5.76-6.78)	6.66 (6.09-7.28)	6.88 (6.28-7.55)
	1	0	1	1	6.57 (6.20-6.97)	6.74 (6.35-7.15)	6.61 (6.29-6.95)	6.58 (6.22-6.96)	6.59 (6.21-6.99)
	0	1	1	1	9.46 (6.02-14.88)	9.65 (6.13-15.18)	8.33 (5.33-13.03)	9.03 (5.82-14.03)	9.61 (6.03-15.3)
4	1	1	1	1	9.45 (8.29-10.78)	9.7 (8.51-11.06)	9.14 (8.10-10.31)	9.19 (8.10-10.43)	9.48 (8.32-10.81)

Sensitivity analysis 1: Additionally adjusted for previous cesareans in the main model.

Sensitivity analysis 2: Multiple imputation for confounding variables

Sensitivity analysis 3: Multiple imputation for both four CVD risk factors and confounding variables

Sensitivity analysis 4: Excluding the underweight subjects (n=1,007,210).
